# Does the virus cross the road? Viral phylogeographic patterns among bobcat populations reflect a history of urban development

**DOI:** 10.1111/eva.12927

**Published:** 2020-02-20

**Authors:** Christopher P. Kozakiewicz, Christopher P. Burridge, W. Chris Funk, Meggan E. Craft, Kevin R. Crooks, Robert N. Fisher, Nicholas M. Fountain‐Jones, Megan K. Jennings, Simona J. Kraberger, Justin S. Lee, Lisa M. Lyren, Seth P. D. Riley, Laurel E. K. Serieys, Sue VandeWoude, Scott Carver

**Affiliations:** ^1^ School of Natural Sciences University of Tasmania Hobart TAS Australia; ^2^ Department of Biology Colorado State University Fort Collins CO USA; ^3^ Graduate Degree Program in Ecology Colorado State University Fort Collins CO USA; ^4^ Department of Veterinary Population Medicine University of Minnesota St Paul MN USA; ^5^ Department of Fish, Wildlife, and Conservation Biology Colorado State University Fort Collins CO USA; ^6^ Western Ecological Research Center U.S. Geological Survey San Diego CA USA; ^7^ Biology Department San Diego State University San Diego CA USA; ^8^ Department of Microbiology, Immunology, and Pathology Colorado State University Fort Collins CO USA; ^9^ Western Ecological Research Center U.S. Geological Survey Thousand Oaks CA USA; ^10^ National Park Service Santa Monica Mountains National Recreation Area Thousand Oaks CA USA; ^11^ Department of Environmental Studies University of California Santa Cruz Santa Cruz CA USA; ^12^ Institute for Communities and Wildlife in Africa Biological Sciences University of Cape Town Cape Town South Africa

**Keywords:** bobcat, connectivity, costructure, feline immunodeficiency virus, genetic, phylogeography, retrovirus, transmission, urbanization

## Abstract

Urban development has major impacts on connectivity among wildlife populations and is thus likely an important factor shaping pathogen transmission in wildlife. However, most investigations of wildlife diseases in urban areas focus on prevalence and infection risk rather than potential effects of urbanization on transmission itself. Feline immunodeficiency virus (FIV) is a directly transmitted retrovirus that infects many felid species and can be used as a model for studying pathogen transmission at landscape scales. We investigated phylogenetic relationships among FIV isolates sampled from five bobcat (*Lynx rufus*) populations in coastal southern California that appear isolated due to major highways and dense urban development. Divergence dates among FIV phylogenetic lineages in several cases reflected historical urban growth and construction of major highways. We found strong FIV phylogeographic structure among three host populations north‐west of Los Angeles, largely coincident with host genetic structure. In contrast, relatively little FIV phylogeographic structure existed among two genetically distinct host populations south‐east of Los Angeles. Rates of FIV transfer among host populations did not vary significantly, with the lack of phylogenetic structure south‐east of Los Angeles unlikely to reflect frequent contemporary transmission among populations. Our results indicate that major barriers to host gene flow can also act as barriers to pathogen spread, suggesting potentially reduced susceptibility of fragmented populations to novel directly transmitted pathogens. Infrequent exchange of FIV among host populations suggests that populations would best be managed as distinct units in the event of a severe disease outbreak. Phylogeographic inference of pathogen transmission is useful for estimating the ability of geographic barriers to constrain disease spread and can provide insights into contemporary and historical drivers of host population connectivity.

## 1 INTRODUCTION

Urban development is a dominant factor influencing wildlife abundance, changes the nature and frequency of both intra‐ and interspecific interactions, and leads to reduced connectivity among populations (Bierwagen, 2007; Crooks, Riley, Gehrt, Gosselink, & Deelen, 2010; Shochat et al., 2010). Thus, urbanization may also alter patterns of pathogen transmission and spatial spread in wildlife (Becker, Streicker, & Altizer, 2015; Fountain‐Jones, Craft, et al., 2017a). This can have consequences for the frequency and severity of disease outbreaks and the probability of pathogen spillover among wildlife, humans and domestic animals (Faust et al., 2018). Most urban wildlife disease research has examined how pathogen prevalence and infection risk is modified by urbanization (Brearley et al., 2013), but relatively few studies investigate the direct impacts of landscape heterogeneity on patterns of pathogen transmission and spread in an urban context. Genetic approaches may be used to understand how heterogeneous landscape factors influence pathogen transmission, with host gene flow often used as a proxy for predicting transmission risk (Kozakiewicz et al., 2018). However, using host gene flow may sometimes be inappropriate because host and pathogen genetic structure can be divergent due to factors such as transmission mode, pathogen reproductive mode or transmission via movement of nonreproducing hosts (Lee et al., 2012; Mazé‐Guilmo, Blanchet, Mccoy, & Loot, 2016; Tesson et al., 2016). Nonetheless, reduced host connectivity due to habitat fragmentation has been shown to reduce rates of spread of diseases such as white‐nose syndrome (Petit & Puechmaille, 2015), while natural barriers such as rivers have been shown to slow the spread of rabies virus (Biek, Henderson, Waller, Rupprecht, & Real, 2007). Understanding the extent to which patterns of pathogen transmission are related to host population structure, as well as the effects of habitat fragmentation on pathogen connectivity, is important for predicting and managing wildlife disease in urban environments.

Bobcats (*Lynx rufus*) are regarded as indicator species for connectivity in urbanizing regions given their sensitivity to anthropogenic disturbance and requirement for connected habitat (Crooks, 2002; Ordeñana et al., 2010). Thus, it follows that directly transmitted bobcat pathogens may similarly be used as a model for understanding disease connectivity in urbanizing environments. Bobcat populations are exposed to a variety of parasites and pathogens (Carver et al., 2016; Riley et al., 2007; Riley, Foley, & Chomel, 2004; Sleeman, Keane, Johnson, Brown, & VandeWoude, 2001), with patterns of spread potentially influenced by landscape connectivity and variation in environmentally or host‐driven selective pressures (Real & Biek, 2007). In addition to the insights into host connectivity that can be derived from directly transmitted pathogens, predicting how future pathogen outbreaks might spread among bobcat populations may also be important from a management perspective (Smith, Acevedo‐Whitehouse, & Pedersen, 2009).

Feline immunodeficiency viruses (FIVs, the feline analogue of HIV) infect many felid species, including bobcats (FIV_Lru_), and have characteristics that make them suitable models for studying pathogen transmission and inferring host movement (Antunes et al., 2008; Biek, Drummond, & Poss, 2006; Fountain‐Jones, Packer, et al., 2017b; Kerr et al., 2018; VandeWoude & Apetrei, 2006). FIVs are directly transmitted and form chronic, lifelong infections, resulting in relatively uncomplicated transmission networks that remain largely intact owing to little overt pathology (Lee et al., 2012; VandeWoude & Apetrei, 2006). FIVs have evolved as species‐specific strains (Pecon‐Slattery, Troyer, Johnson, & O’Brien, 2008), with the only known examples of cross‐species transmission occurring from bobcats to puma and a single recorded domestic cat to puma event (Franklin et al., 2007; Lee et al., 2017). The FIV genome mutates rapidly, and FIV proviral DNA can be readily isolated from host blood or tissue (Biek et al., 2003). Using Bayesian phylogenetic approaches, recent transmission histories of rapidly evolving viruses can be inferred, including temporal estimates of divergence among virus populations (Drummond, Nicholls, Rodrigo, & Solomon, 2002). Patterns of FIV phylogeographic structure may indicate variation in rates of viral transmission and spread. Such variation can arise through altered connectivity or local adaptation of the pathogen to environmental or host immunological factors (Kaltz & Shykoff, 1998; Lion & Gandon, 2015; Real & Biek, 2007). In addition, because increased isolation of host populations would likely decrease introduction of novel viral variants and place constraints on viral population sizes due to a limited number of potential hosts, we might expect reduced viral diversity in more isolated populations (e.g. Fountain‐Jones, Craft, et al., 2017a). Thus, genetic analysis of FIVs can elucidate how urban habitat fragmentation, through its impacts on host connectivity, may influence the spread of directly transmitted diseases.

**Figure 1:**
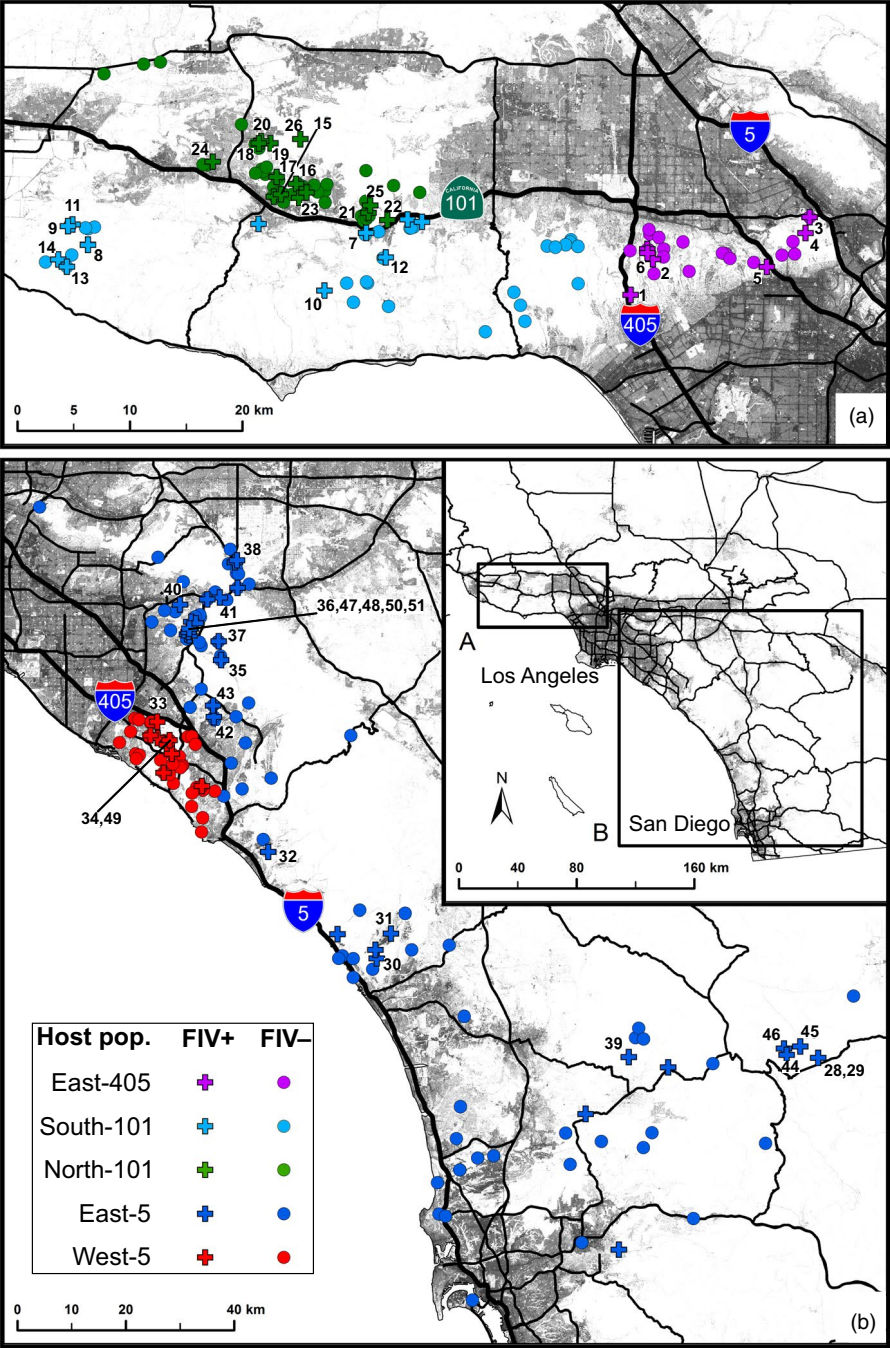
Five distinct bobcat populations in coastal southern California are infected with FIV_Lru_: (a) north‐west of Los Angeles and (b) south‐east of Los Angeles. Grey shading indicates urban development, including the cities of Los Angeles and San Diego (see inset). Black lines indicate highways, with highways acting as barriers to gene flow among host populations labelled with route markers and indicated as bold black lines. Marker colours indicate host population, with crosses indicating FIV‐positive individuals and circles indicating FIV‐negative individuals. All FIV‐positive samples included in phylogenetic analysis are numbered for cross‐referencing with Figure 2. FIV‐positive samples that lack numbers indicate unsuccessful genotyping

Directly transmitted, species‐specific pathogens such as FIV_Lru_ carry the expectation that their spread is closely tied to factors influencing host connectivity (McCallum, 2008). Several genetically distinct bobcat populations exist north‐west and south‐east of Los Angeles, California, with major freeways and large urban areas forming the primary barriers to gene flow (Lee et al., 2012; Poessel et al., 2014; Riley et al., 2006; Ruell et al., 2012; Serieys, Lea, Pollinger, Riley, & Wayne, 2015). Increasing urbanization in this region is also generally further reducing connectivity within populations (Kozakiewicz et al., 2019). However, analysis of FIV_Lru_ may provide insights into both host and pathogen connectivity that are undetectable using host genetics or telemetry studies alone. The Interstate 5 (Figure 1) is one of the largest freeways in this region and has been shown to form a substantial barrier to bobcat gene flow, but it has also been shown to be permeable to FIV_Lru_ transmission (Fountain‐Jones, Craft, et al., 2017a; Lee et al., 2012). This discordance between host gene flow and FIV_Lru_ transmission indicates that bobcats are still crossing Interstate 5 but likely not reproducing, reflecting greater sensitivity of the viral genetic patterns to host movements than host genetic patterns; that is, opportunities for viral transmission exist that do not necessitate host reproduction. However, despite evidence for FIV_Lru_ transmission across Interstate 5, FIV_Lru_ genetic diversity and evolutionary rates differed among populations (Fountain‐Jones, Craft, et al., 2017a), which may point to varying patterns of selection. These prior genetic studies on bobcats and their pathogens have focused on a small region of southern California, on a single barrier and on a relatively small number of samples. Further work is required to test the generality of discordance between host connectivity and transmission with respect to major urban barriers and to elucidate the mechanisms driving this relationship.

Here, we test whether patterns of viral genetic structure are consistent with known anthropogenic barriers to host gene flow, and whether viral genetic structure can provide insights into host movements that are not detected from host genetic analysis. To address this, we sequence FIV_Lru_ isolated from fragmented bobcat populations across coastal southern California (from north of Los Angeles to San Diego). Implementing Bayesian viral phylogeography in discrete space, we quantify FIV_Lru_ connectivity and assess the permeability of several major highway and urban barriers to pathogen spread. In doing so, we aim to understand whether viral phylogenetic patterns reflect the expansion of urban development in one of the most rapidly urbanizing landscapes in North America. We predict weak FIV_Lru_ phylogeographic structure with respect to host populations, and thus, that FIV_Lru_ transmission is occurring among bobcat populations in this region despite minimal host gene flow between habitat fragments. We also expect that more genetically isolated bobcat populations will have a lower diversity of FIV_Lru_ strains and may exert differential selection pressure on FIV_Lru_ relative to the less‐isolated populations. This study provides novel insights into host and pathogen connectivity with respect to major urban barriers.

## 2 METHODS

### 2.1 Study area, sample collection and DNA extraction

Blood (*n* = 265) and tissue (*n* = 27) samples were collected from 292 bobcats in three adjoining areas in coastal southern California (Figure 1). Forty‐five were collected from north and east of San Diego between 2007 and 2012 (Jennings & Lewison, 2013), 113 were collected from south‐east of Los Angeles between 2002 and 2010 (Lyren, Alonso, Crooks, & Boydston, 2008a, 2008b; Lyren et al., 2006), and 134 were collected from north‐west of Los Angeles between 1997 and 2011 (Riley et al., 2006; Serieys et al., 2015). Collectively, these samples represent five genetically distinct bobcat populations separated by major highways and urban areas that pose barriers to host gene flow (Kozakiewicz et al., 2019). We label these populations according to their location with respect to major highway barriers: North‐101, South‐101, East‐405, West‐5 and East‐5 (Figure 1). All samples were derived from either live trapping or opportunistically collected roadkill, where sample dates and locations were recorded. Sex was recorded at the time of capture, and age (juvenile < 2 years; adult > 2 years) was estimated according to size, weight and dentition. All live animals were captured, handled and released according to protocols approved by cooperating agencies and relevant animal ethics authorities (for detailed information, see Jennings & Lewison, 2013; Lyren, Alonso, Crooks, & Boydston, 2008a; Lyren, Alonso, Crooks, & Boydston, 2008b; Lyren et al., 2006; Riley et al., 2006; Serieys et al., 2015). We extracted DNA using the Qiagen DNeasy Blood & Tissue Kit (Qiagen Inc.) according to the manufacturer's instructions.

### 2.2 FIV screening and amplification

Bobcats infected with FIV_Lru_ were identified using a PCR assay targeting proviral DNA. We used nested PCR primers designed by Lee et al. (2014) to amplify 547 bp from a region of the FIV *gag* gene. This region is highly conserved and was chosen to reduce the probability of false negatives due to polymorphism at primer sites. First‐round screening primers were A6F and A9R, and second‐round screening primers were A7F and A8R (Lee et al., 2014). Screening PCRs were conducted in 50 μl reactions using Invitrogen Platinum Taq DNA Polymerase (Thermo Fisher Scientific), with first‐round reactions containing 5 μl of DNA, which varied in concentration among samples between 10 and 100 ng/μl. Second‐round reactions contained 2 µl of first‐round PCR product. Reaction conditions included 94°C for 2 min and 13 cycles of the following: 94°C for 30 s, touchdown annealing at 56°C–50°C (first round) or 59°C–53°C (second round) for 30 s and decreasing 0.5°C every cycle, and an extension at 72°C for 30 s. These were followed by a further 27 cycles with the same conditions as the previous cycle. A final extension temperature of 72°C was held for 5 min. PCRs were conducted in small batches of 16 to minimize risk of contamination, and each batch included two positive controls and one negative control. PCR product was visualized under UV light on a 0.7% agarose gel containing Gel Red (Gold Biotechnology Inc.). We identified a total of 73 FIV‐positive bobcats, using both blood and tissue samples, which we included in FIV_Lru_ genetic analysis below. We used a logistic regression with a likelihood‐ratio test (LRT) to explore trends in FIV_Lru_ prevalence among populations. A second logistic regression was used to test for differences in FIV_Lru_ prevalence among sexes and age categories, to assess variation in FIV_Lru_ prevalence over the sampling period, and to identify differences in detectability between blood and tissue samples.

To investigate phylogenetic relationships among FIV_Lru_ samples, we analysed a region of the highly variable *env* gene encompassing the entire domain encoding the transmembrane protein and approximately half the domain encoding the surface protein (Lee et al., 2014). This region was chosen due to its high polymorphism and documented use for phylogenetically inferring recent FIV transmission at fine spatial scales (Biek et al., 2006, 2003; Fountain‐Jones, Craft, et al., 2017a; Lee et al., 2012). PCR amplification of a 1.6‐kb fragment spanning a portion of the *env* gene to the 3’ long terminal repeat (LTR) was conducted using nested PCR primers and protocols developed by Lee et al. (2014). First‐round primers were A21F and A32R, which amplified a 2,267‐bp fragment. Second‐round primers were A22F and A31R, which amplified a 1,547‐bp fragment. PCR was conducted in 50 μl reactions using Invitrogen Platinum Taq DNA Polymerase High Fidelity, with first‐round reactions containing 5 μl of DNA as above, and second‐round reactions containing 2 μl of first‐round PCR product. First‐round reaction conditions included 94°C for 1 min, 13 cycles comprising melting at 94°C for 30 s, touchdown annealing at 58°C–52°C for 30 s and decreasing 0.5°C every cycle, and extension at 68°C for 2.5 min. These were followed by a further 27 cycles with the same conditions except annealing at 52°C and then final extension at 68°C for 3 min. Second‐round reaction conditions included 94°C for 1 min followed by 17 cycles comprising melting at 94°C for 30 s, touchdown annealing at 59°C–51°C for 30 s and decreasing 0.5°C every cycle, and extension at 68°C for 1.5 min. These were followed by a further 23 cycles with the same conditions except annealing at 51°C and a final extension at 68°C for 3 min.

Following second‐round reactions and verification of amplification using gel electrophoresis as above, PCR products were purified using ExoSAP‐IT PCR Product Cleanup Reagent (Thermo Fisher Scientific) according to the manufacturer's protocol. Purified PCR products were sequenced at Macrogen USA on an ABI 3730xl using internal forward and reverse primers (A23F and A23R; Lee et al., 2014) in addition to second‐round PCR primers above (A22F and A31R; Lee et al., 2014).

### 2.3 Sequence analysis and alignment

All visualization and manipulation of sequence chromatograms were conducted using Geneious version 11.1.4. We manually screened all chromatograms to ensure they were correctly scored. We constructed a consensus of 13 existing Californian bobcat FIV_Lru_ sequences from GenBank (Lee et al., 2014; accession numbers KF906143.1–KF906155.1) and mapped all reads to this, forming a new consensus sequence for each individual. Some FIV_Lru_ genetic variation within hosts was observed, indicated by multiple chromatogram peaks at the same base position across multiple reads. In such cases, the “dominant” variant was scored, identified as the highest quality peak at a given base position. If this method was unable to resolve the dominant genotype, the position was scored using the appropriate IUPAC degenerate character.

We used “Find ORFs” in Geneious to identify the *env* open reading frame (ORF) for each sequence. All sequences were trimmed to include only *env* to a final length of 1,257 bp, removing the sequenced 3′ LTR. Stop codons were removed from the end of all ORFs, and sequences were aligned using the MUSCLE translation alignment. The final alignment included sequences genotyped in this study together with 13 GenBank sequences trimmed from full genomes sequenced by Lee et al. (2014; accession numbers as above).

Aligned ORFs were examined for recombination breakpoints using RDP version 4.96 (Martin, Murrell, Golden, Khoosal, & Muhire, 2015) with several recombination detection methods and assessment of consensus among methods. Recombination detection methods used were RDP (Martin & Rybicki, 2000), GENECONV (Padidam, Sawyer, & Fauquet, 1999), Chimaera (Posada & Crandall, 2001), MaxChi (Maynard Smith, 1992), BootScan (Salminen, Carr, Burke, & McCutchan, 1995), SiScan (Gibbs, Armstrong, & Gibbs, 2000) and 3Seq (Boni, Posada, & Feldman, 2007), with recombination breakpoints accepted if detected using more than three of these methods at a significance of *p* < .05. Any recombinant regions were removed for subsequent analysis. To quantify genetic diversity among FIV_Lru_ strains within host populations, nucleotide diversity (*π*) was estimated using DnaSP (Rozas et al., 2017).

### 2.4 Phylogenetic analysis

We constructed a temporally explicit Bayesian phylogeny using a 1,257‐bp region of the FIV_Lru_ *env* gene with Bayesian Evolutionary Analysis Sampling Trees (BEAST) version 1.10.4, using BEAUti to construct the input files (Drummond, Suchard, Xie, & Rambaut, 2012). Tree tip dates were specified according to sampling date. To ensure that there was sufficient temporal signal for estimation of virus divergence dates, we used the R package TipDatingBeast (Rieux & Khatchikian, 2017) to perform date randomization tests for temporal signal and use leave‐one‐out cross‐validation to identify sequences potentially biasing temporal estimates (Duchêne, Duchêne, Holmes, & Ho, 2015).

After confirming temporal signal, we tested several BEAST models comprising different substitution models and molecular clocks and selected the most appropriate using marginal likelihood estimation with path and stepping‐stone sampling (Baele et al., 2012). The highest supported model included the HKY substitution model with gamma‐distributed rate heterogeneity and a proportion of invariant sites, the CP_112_ codon partition model, and a lognormal uncorrelated relaxed molecular clock (Supporting Information 1: Table S1). We specified the Gaussian Markov random field Bayesian skyride tree prior (Minin, Bloomquist, & Suchard, 2008) and used default settings for all other priors and operators. To estimate ancestral host populations of FIV_Lru_ and quantify FIV_Lru_ exchange among host populations, we performed a discrete space phylogeographic analysis as part of the BEAST phylogenetic reconstruction above. Specifying host population as a discrete trait, we used an asymmetric discrete trait substitution model with a Bayesian stochastic search variable selection procedure to estimate rates of transition between host populations, and we reconstructed host population states at all ancestors. One sequence from north‐west of Los Angeles for which the host population was unknown was assigned an ambiguous state that could be one of North‐101, South‐101 or East‐405. We estimated Bayes factor support for transition rates using SpreaD3 (Bielejec et al., 2016), which we evaluated according to Kass and Raftery (1995). We ran three sets of 100 million Markov chain Monte Carlo iterations, sampling every 10,000 iterations and excluding the initial 10% of each set as burn‐in. Model convergence was checked and parameters were evaluated, ensuring an effective sample size > 200, using Tracer version 1.7 (Rambaut, Drummond, Xie, Baele, & Suchard, 2018). A maximum clade credibility tree was constructed from the sampled trees using TreeAnnotator version 1.10 from the BEAST package and visualized using FigTree version 1.4.3 (http://tree.bio.ed.ac.uk/software/figtree/).

To complement the above phylogeographic analysis and further investigate the extent to which rates of FIV_Lru_ transition varied among different pairs of host populations, we tested phylogenetic models of rate heterogeneity of FIV_Lru_ host population shifts using *geiger* (Harmon, Weir, Brock, Glor, & Challenger, 2008) in R (R Development Core Team, 2013). In these models, as above, we treated host population as a discrete trait, with transitions among trait states being evidence for FIV_Lru_ spread from one host population to another. We fitted three models that tested different hypotheses regarding relative rates of trait state change along the virus phylogeny: (a) all transitions among states occur at the same rate (null model); (b) all transitions among states occur at different rates; and (c) all transitions among states occur at different rates but are symmetrical (e.g. transitions from state 1 to state 2 occur at the same rate as transitions from state 2 to state 1, but at a different rate from transitions involving other states). We conducted likelihood‐ratio tests of support for each variable‐rate hypothesis in explaining the observed distribution of transitions among host populations across our virus maximum clade credibility tree, relative to the null model. These hypotheses were tested both before and after removal of branches undergoing selection to ensure that the spatial distribution of selection was not producing false signals of homogeneity (or heterogeneity) in road crossings.

Although we were predominantly interested in spatial variation in FIV_Lru_ connectivity as a driver of phylogenetic patterns, phylogeographic structure in pathogens can occur due to local adaptation, arising from spatial variation in host immunity or environmental conditions that can affect the fitness of viral lineages (Lion & Gandon, 2015). For example, differences among host populations may result in positively selected pathogen lineages that are specific to the host populations to which they have adapted. Conversely, multiple lineages undergoing positive selection can also exhibit convergent evolution and thus closer phylogenetic relatedness than their ancestry alone might predict (Edwards, 2009). To identify signatures of positive selection acting across branches, we used the BUSTED (Murrell et al., 2015) method implemented in HyPhy (Pond, Frost, & Muse, 2005), based on the maximum clade credibility tree generated above. We tested all branches using aBSREL (Smith et al., 2015) implemented in HyPhy to identify specific branches upon which positive selection is acting, using a Holm–Bonferroni‐corrected *P*‐value cut‐off of 0.05.

## 3 RESULTS

In total, we tested 292 bobcats for FIV_Lru_ and found an overall prevalence of 25.0% (Figure 1). Prevalence did not vary significantly among five genetically distinct host populations (21.7%–27.3%; logistic regression LRT: *G*_1_ = 0.72, *p* = .94). FIV_Lru_ infection was more likely among males than females (*G*_1_ = 10.01, *p* < .01), and less likely among young individuals than adults (*G*_1_ = 6.90, *p* < .01). Likelihood of FIV_Lru_ infection did not change with respect to sample date (*G*_1_ = 2.07, *p* = .15) or sample type (i.e. blood or tissue; *G*_1_ = 1.16, *p* = .28). Five individuals screened for FIV_Lru_ lacked precise location data, including one FIV_Lru_‐positive individual, and were thus not included in the above estimates.

We obtained a total of 53 sequences for a 1,257‐bp region of the FIV *env* gene, including the aforementioned 13 previously published sequences (Lee et al., 2014). We were unable to obtain sequences of sufficient quality for 20 FIV_Lru_‐positive samples, likely due to DNA degradation, low FIV_Lru_ copy number, the presence of GC rich regions or mutations at primer binding sites. Sequencing success did not appear to vary spatially or temporally. In addition, two sequences sampled from siblings at similar locations were identical, so only one was included in analyses for a total of 52. Mean pairwise sequence identity was 89.7%. Recombination was not detected within the 1,257‐bp region despite previous evidence for recombination in this gene (Lee et al., 2014), likely due to the relatively small fragment size and the focus of this study on a single geographic region.

### 3.1 Phylogenetic analysis

**Figure 2:**
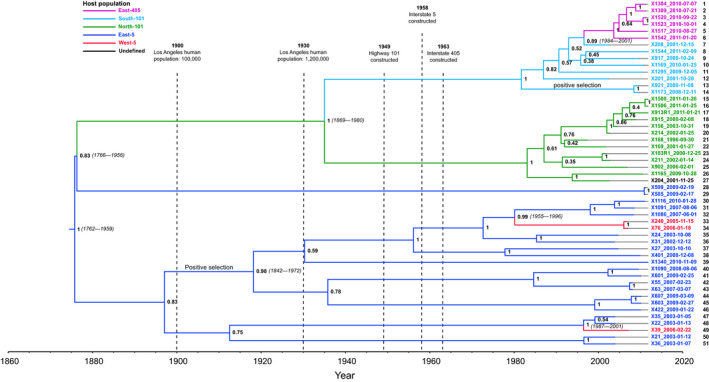
FIV_Lru_ exhibits phylogeographic structure with respect to bobcat host populations, with divergence dates reflecting decreasing connectivity as urbanization around Los Angeles has increased. Maximum clade credibility Bayesian phylogenetic tree was constructed using 1,257 nt sequences of the env gene region in FIV_Lru_ sampled from bobcats in coastal southern California, with dated tips. Node labels indicate posterior probabilities, with 95% highest posterior density estimates indicating confidence of divergence date estimates shown in parentheses for nodes associated with among‐population transmission. Branches inferred to be undergoing positive selection are labelled as such. Tip label colours indicate the host population from which a given sample was collected, and branch colours indicate reconstructed ancestral host populations. Dates of major urban features corresponding to host population structure are shown with vertical dashed lines. All tips are numbered for cross‐referencing with sample locations in Figure 1

We identified and subsequently removed one sequence as significantly biasing temporal estimates (Supporting Information 1 Figure S1), leaving a total of 51 sequences for phylogenetic analysis. Bayesian phylogenetic analysis indicated two major clades corresponding to FIV_Lru_ isolates from either north‐west of Los Angeles (North‐101, South‐101 and East‐405 populations) or south‐east of Los Angeles (East‐5 and West‐5 populations; Figure 2). These two lineages were estimated to have diverged from a common ancestor in approximately the year 1875 (95% highest posterior density; *HPD* = 1762–1959; Table 1), and this node had high posterior support (*PP* = 1.00). The clade identified south‐east of Los Angeles was characterized by relatively deeper divergences among isolates, with many long branches comprising relatively few isolates each. The earliest of these divergences likely occurred prior to 1900 (*PP* = 0.83). The two largest subclades south‐east of Los Angeles likely diverged around 1918 (95% *HPD* = 1842–1972) with high posterior support (*PP* = 0.98).

**Table 1:** Among‐host‐population phylogeographic divergences of bobcat feline immunodeficiency virus and potentially associated urban influences

Estimated year	Divergence event
1875 (1762–1959)	FIV strains from north‐west of Los Angeles diverged from strains south‐west of Los Angeles. City of Los Angeles human population approaching 100,000 (U.S. Census Bureau, 1900)
1876 (1766–1956)	An additional lineage containing two contemporary isolates sampled south‐east of Los Angeles diverged from the group north‐west of Los Angeles. City of Los Angeles human population approaching 100,000 (U.S. Census Bureau, 1930)
1934 (1869–1980)	Divergence among lineages sampled north of highway 101 and those sampled south of 101. Construction of the 101 was completed in 1949
1980 (1955–1996)	First West‐5 lineage diverged from East‐5 population, an estimated 22 years after the Interstate 5 was constructed in 1958
1996 (1987–2001)	Second West‐5 lineage diverged from East‐5 population
1997 (1984–2001)	All contemporary isolates sampled east of the Interstate 405 diverged from those sampled west of Interstate 405, an estimated 34 years after the 405 was constructed in 1963

Note

95% high posterior density intervals for divergence date estimates shown in parentheses.

Within the clade south‐east of Los Angeles, there was an absence of phylogeographic structure in FIV_Lru_ with respect to host population boundaries or other geographic features, although geographically proximal isolates were sometimes closely related (e.g. samples numbered 44–46; Figures 1b, 2). Two FIV_Lru_ isolates from the West‐5 population (samples 33, 34) were more closely related to each other than to isolates from East‐5. These West‐5 isolates diverged from East‐5 isolates in approximately 1980 (95% *HPD* = 1955–1996; *PP* = 0.99). Another divergence among West‐5 and East‐5 isolates occurred in approximately 1996 (95% *HPD* = 1987–2001; *PP* = 1.00).

In contrast to the clade south‐east of Los Angeles, FIV_Lru_ isolates sampled north‐west of Los Angeles show strong phylogeographic structure with respect to host populations. This clade forms two major groups that diverged, with high posterior support (*PP* = 1.00), in approximately 1934 (95% *HPD* = 1869–1980). This divergence occurred between a group comprising all isolates sampled from the South‐101 and East‐405 populations and another group containing all FIV_Lru_ isolates sampled from the North‐101 population. All East‐405 samples form a single clade that diverged from South‐101 in approximately 1997 (95% HPD = 1984–2001). Two isolates sampled from south‐east of Los Angeles (28, 29) diverged from the north‐west LA group separately from the other south‐east samples but at approximately the same time—around 1876 (95% *HPD* = 1766–1956; *PP* = 0.83). These two isolates (from East‐5) were sampled from similar locations at the far eastern extent of our study area and may represent a distinct geographic group.

Discrete trait transition (i.e. transmission among populations) rates were the highest for transitions from the East‐5 host population to West‐5 (1.36) and from South‐101 to East‐405 (1.31), with both of these rates receiving decisive Bayes factor support. We found substantial support for a transition rate of 1.09 between North‐101 and South‐101, and of 0.90 between East‐5 and North‐101, which was the lowest transition rate across all pairs of populations. All other pairs of populations had poorly supported transition rates ranging between 0.91 and 1.03 (Supporting Information 1 Table S2). In contrast, likelihood‐ratio tests of highway crossing rate models indicated no support for either variable symmetrical rate (i.e. highway crossing rates are the same in both directions but may vary among pairs of host populations; *p* = .50, *x* ^2^ = 12.4, *df* = 19) or variable asymmetrical rate (i.e. highway crossings rates are different in each direction and may vary among pairs of host populations; *p* = .87, *x* ^2^ = 8.32, *df* = 9) state change models over the equal rate state change model (i.e. highway crossing rates do not vary among host populations). Tests following removal of branches undergoing selection produced similar results. Thus, despite increased transition rates being evident among some pairs of populations, overall, the observed patterns of FIV_Lru_ spread across urban barriers are best explained by equal rates of FIV_Lru_ exchange among each pair of host populations.

### 3.2 Sequence diversity and selection

**Figure 3:**
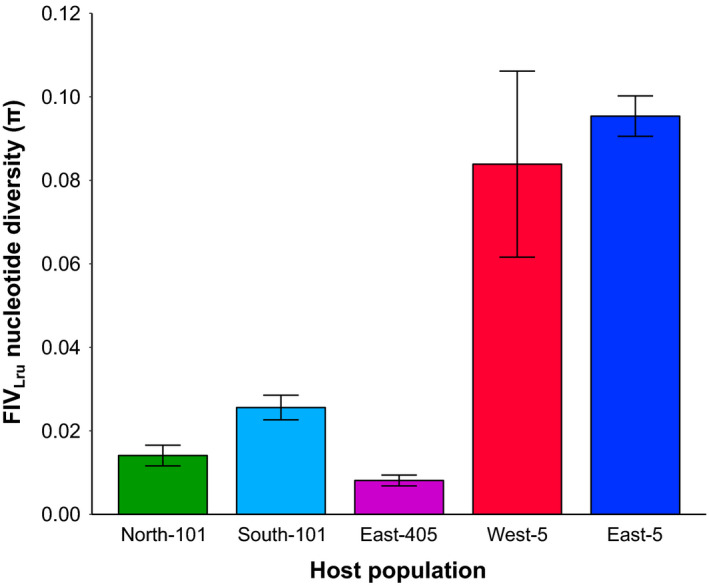
FIV nucleotide diversity (±*SD*) is higher in bobcat populations located south‐east of Los Angeles (West‐5 and East‐5) than those located north‐west of Los Angeles (North‐101, South‐101, and East‐405)

Although FIV_Lru_ prevalence was similar among regions, FIV_Lru_ sequences were most diverse south‐east of Los Angeles (Figure 3), with East‐5 (*π* = 0.095; *SD* = 0.005) and West‐5 (*π* = 0.084; *SD* = 0.022) having higher nucleotide diversity than North‐101 (*π* = 0.014; *SD* = 0.002), South‐101 (*π* = 0.026; *SD* = 0.003) and East‐405 (*π* = 0.008; *SD* = 0.001). BUSTED analysis showed evidence for positive selection acting on this gene region (*p* < .0001). aBSREL tested all 103 branches for evidence of positive selection and identified two branches showing signatures of selection across the sequence (Figure 2). One of these branches contained only two FIV_Lru_ isolates from the South‐101 population that was paraphyletic to all other isolates sampled from that population. The other branch showing evidence for positive selection was the large branch comprising a majority of the FIV_Lru_ sampled from south‐east of Los Angeles that diverged into two large subclades.

## 4 DISCUSSION

We conducted a spatially intensive viral phylogeographic study in an urban environment, enabling us to assess phylogeographic patterns in FIV_Lru_ among several host populations and infer how historical patterns of urbanization have potentially influenced bobcat connectivity over time. We found distinct differences in FIV_Lru_ phylogenetic structure among populations in different regions in coastal southern California. In general, populations south‐east of Los Angeles had greater divergence among FIV_Lru_ lineages, had higher FIV_Lru_ genetic diversity and lacked clear phylogeographic structure. In contrast, the populations north‐west of Los Angeles were relatively more isolated, with all FIV_Lru_ isolates sampled north of highway 101 forming a single clade and those sampled south of highway 101 forming another clade comprising the South‐101 population from which the East‐405 population later descended. The reduced diversity of FIV_Lru_ populations north‐west of Los Angeles suggests that each population descended from relatively fewer FIV_Lru_ strains than in the south‐west populations, with little subsequent diversification within each population potentially as a result of negative selection and genetic drift.

The observed phylogenetic relationships provide evidence that major highways are limiting FIV_Lru_ connectivity among bobcat populations around Los Angeles. Despite marked differences in phylogenetic structure north‐west and south‐east of Los Angeles, we found that rates of FIV_Lru_ exchange across Interstate 5 (south‐east of Los Angeles) were not substantially higher than those observed for Interstate 405 (north‐west of Los Angeles) and did not vary significantly across all pairs of populations. Instead, the greater number of inferred highway crossings south‐east of Los Angeles may be due to the greater depth of branches here compared with north‐west of Los Angeles, thus having a greater probability of observing crossings over this longer time frame. On the other hand, the low number of samples from the West‐5 population may have limited our power to detect differences in discrete trait transition rates. Compared with populations north‐west of Los Angeles, the East‐5 FIV_Lru_ population has substantially higher genetic diversity and a higher population size owing to a large host population size and its openness to unsampled populations to the east that may serve as potential sources for diverse FIV_Lru_ strains (Kozakiewicz et al., 2019). This is perhaps illustrated by the two samples near the eastern edge of our study area that form a lineage that is entirely distinct from all others in the East‐5 population, having diverged separately from the populations north‐west of Los Angeles in approximately 1876. The relatively higher exposure of the East‐5 population to novel FIV_Lru_ strains suggests potentially increased susceptibility of these populations to other novel, potentially more virulent pathogens compared with the more isolated populations north‐west of Los Angeles. However, the conservation implications of this exposure are likely tempered by the beneficial effects of increased connectivity on host genetic diversity and subsequently increased resilience to stressors such as disease (McCallum & Dobson, 2002).

FIVs are directly transmitted, so movements of FIV strains reflect the movements of infected hosts. Thus, by phylogenetically estimating divergence dates among virus strains, we can infer how historical patterns of urbanization have potentially influenced bobcat connectivity over time. Estimated divergence dates with respect to major anthropogenic barriers generally reflected the chronology of urban development in coastal southern California (Table 1). The divergence of the two major FIV_Lru_ clades estimated at around 1875 coincides roughly with the city of Los Angeles’ initial development boom when its human population expanded from ~6,000 to 100,000 between 1,870 and 1,900 (U.S. Census Bureau, 1900). We found no evidence of further transmission between north‐west and south‐east of Los Angeles since ca. 1876. It is unlikely that at this time, the extent of urban development in the Los Angeles Basin was alone sufficient to preclude movement of bobcats among the areas inhabited by the two clades we identified. However, a large proportion of anthropogenic land use associated with Los Angeles at this time consisted of agriculture (Nelson, 1959), which would also have contributed to loss of suitable bobcat habitat and has been suggested to reduce connectivity in contemporary populations (Hilty & Merenlender, 2004; Nogeire et al., 2015).

Highway 101 is a primary driver of host population structure in bobcats north‐west of Los Angeles (Riley et al., 2006; Serieys et al., 2015), and its completion in 1949 corresponds to a divergence in FIV_Lru_ estimated to have occurred in ~1935. This suggests that Highway 101 has constituted a major barrier to FIV_Lru_ transmission since and possibly before its completion, with no evidence in contemporary populations of any FIV_Lru_ strains having crossed this road. In contrast, previous research has indicated some highway crossing by bobcats, particularly from south to north, evidenced from both genetic population assignment and telemetry (Riley et al., 2006), with subsequent support from genetic studies (Kozakiewicz et al., 2019; Serieys et al., 2015). In theory, these movements should facilitate FIV_Lru_ transmission, which is thought to occur predominantly through aggressive or sexual contact (Courchamp, Say, & Pontier, 2000). However, coalescent simulation has suggested bobcats that cross highway 101 rarely reproduce (Riley et al., 2006), potentially indicating that immigrants are infrequently capable of sexually transmitting FIV_Lru_. Nonetheless, in a territorial species such as bobcat, we might also expect aggressive interactions to be more frequent where immigrants are involved. Thus, it remains unclear precisely why transmission across highways north‐west of Los Angeles appears to be occurring infrequently relative to rates of host movement.

All six isolates sampled from the East‐405 population diverged from the majority of isolates in the South‐101 clade in ~1997, providing evidence that the Interstate 405 has acted as a barrier to FIV_Lru_ transmission in addition to host gene flow (Kozakiewicz et al., 2019; Serieys et al., 2015). The Interstate 405 was completed in 1962, and the 95% HPD intervals for this divergence estimate only extend back to 1984. It is therefore probable that transmission occurred among the South‐101 and East‐405 populations for at least three decades following the completion of the Interstate 405, with bobcats potentially utilizing existing underpasses under Interstate 405. Host genetic studies also provide evidence of some movement across Interstate 405 (Kozakiewicz et al., 2019; Serieys et al., 2015), providing further indication of potential for FIV_Lru_ spread, though apparently of insufficient frequency for multiple FIV_Lru_ lineages of South‐101 origin to establish in East‐405. We note that throughout our analysis, the 95% highest posterior density intervals are broad and reflect a degree of uncertainty in the estimated divergence dates. However, given the dramatic impact that highways and intense urbanization have on wildlife connectivity, and the lack of other known barriers in the region (e.g., rivers), we believe it likely that highways/development are leading to the observed patterns.

The other major barrier known to be driving bobcat host population structure is the Interstate 5 south‐east of Los Angeles (Kozakiewicz et al., 2019; Lee et al., 2012; Poessel et al., 2014; Ruell et al., 2012). Our results suggest that despite little visible phylogeographic structure with respect to these host populations, rates of FIV_Lru_ exchange are comparable to those among more highly structured populations north‐west of Los Angeles. However, we did observe two lineages of FIV_Lru_ in the West‐5 population that originated in the East‐5 population, diverging in ~1980 and 1996, consistent with previous studies in this area (Fountain‐Jones, Craft, et al., 2017a; Lee et al., 2012). These events occurred following the completion of Interstate 5 in 1958, suggesting that some transmission across this highway has occurred since its completion. Several other large highways run through our study area south‐east of Los Angeles, including Interstate 15, which constitutes a barrier to gene flow in mountain lions (Ernest, Vickers, Morrison, Buchalski, & Boyce, 2014) but apparently not bobcats (Kozakiewicz et al., 2019). Concordantly, we observed no FIV_Lru_ phylogeographic structure with respect to these highways.

Spatial heterogeneity in either environmental factors or host immunology may create variation in conditions to which pathogens must adapt and may influence pathogen phylogeographic patterns due to, for example, higher fitness of locally adapted strains resulting in increased pathogen population structure (Kaltz & Shykoff, 1998). The transmembrane and surface protein constituents of *env* serve a protective function, bind the host cell receptor and are targeted by host antibodies. Thus, this region is thought to be under relatively high immunological selection pressure from the host, containing 12 amino acid sites evolving under positive selection (Lee et al., 2014). We explored the potential of local adaptation to explain the observed phylogeographic patterns and found no evidence of positive selection driving patterns of FIV_Lru_ population structure north‐west of Los Angeles. However, we did find evidence for positive selection acting on two lineages, including a branch that diverged into two large clades comprising a majority of FIV_Lru_ isolates from south‐east of Los Angeles. These clades do not reflect any clear spatial pattern but do encompass a large geographic area containing a large, genetically diverse host population (Kozakiewicz et al., 2019). Although it is unclear what factors are driving this signal of selection, variation in host immunity across the south‐east area may be generating selective pressure on FIV_Lru_ lineages circulating here.

In conclusion, our results demonstrate that the degree of congruence among host and pathogen spatial population structure can vary among populations with comparable landscape characteristics. However, we show that where incongruence occurs, this does not necessarily indicate substantially increased rates of pathogen transmission among host populations. In general, FIV_Lru_ rarely crosses major barriers to host connectivity, and the rate at which this occurs does not appear to vary significantly among regions. This infrequent crossing suggests that major highways and dense urban development constrain the spread of directly transmitted pathogens in bobcats. Thus, the management of more virulent pathogens could benefit from strategies that consider these populations as distinct units. Further, the utility of these highways as barriers to disease spread can be enhanced by targeting surveillance and interventions to known crossing locations such as culverts and underpasses. Direct assessment of pathogen transmission is important for estimating the ability of geographic barriers to constrain disease outbreaks. However, pathogen phylogenetic structure (or lack thereof) should be carefully scrutinized before conclusions are drawn about rates of transmission among contemporary host populations.

## CONFLICT OF INTEREST

None declared.

## Data Availability

All sequence data have been made available on NCBI GenBank. GenBank accession numbers are listed in Supporting Information 2.
