# Corrigendum

**DOI:** 10.1111/eva.13171

**Published:** 2020-12-07

In the article by Gooley et al., the funding information in acknowledgement section has been updated from “G.T. was supported in part by funding provided through the Smithsonian Institution's Short‐Term Visitor Fellowship program” to “G.T. was supported in part by funding provided through the Smithsonian Institution's Short‐Term Visitor Fellowship program and by funding for the project #51148284 from Saint Petersburg State University, Saint Petersburg, Russia.”
