# Supplementary material for: Does the virus cross the road? Viral phylogeographic patterns among bobcat populations reflect a history of urban development
Source: Evol Appl. 2020 Feb 20;13(8):1806–17. doi: 10.1111/eva.12927 (PMC7463333; doi:10.1111/eva.12927)
Supplement: Supplementary file 1 [file EVA-13-1806-s001.pdf]

Supporting Information 1

Does the virus cross the road? Viral phylogeographic patterns among bobcat populations reflect a history of urban development

Authors: Christopher P. Kozakiewicz, Christopher P. Burridge, W. Chris Funk, Meggan E. Craft, Kevin R. Crooks, Robert N. Fisher, Nicholas M. Fountain-Jones, Megan K. Jennings, Simona J. Kraberger, Justin S. Lee, Lisa M. Lyren, Seth P.D. Riley, Laurel E.K. Serieys, Sue VandeWoude, Scott Carver

Tables

Table S1. BEAST model selection results for tests of substitution models and molecular clocks. Log-maximum likelihood scores for path sampling (PS) and stepping-stone sampling (SS) are shown.

Substitution model	Molecular clock	Log ML (PS)	Log ML (SS)
GTR	strict	-8457.96	-8457.72
GTR	uncorrelated relaxed	-8354.26	-8354.10
HKY	strict	-8477.64	-8477.39
HKY	uncorrelated relaxed	-8351.76	-8352.04

Table S2. Continuous time Markov chain transition rates of FIV_{Lru} among pairs of bobcat host populations. BF: Bayes factor.

From	To	Transition rate	BF
East-405	East-5	0.93	1.15
East-405	North-101	0.93	1.21
East-405	South-101	0.94	1.16
East-405	West-5	0.93	1.06
East-5	East-405	0.93	0.52
East-5	North-101	0.90	3.28
East-5	South-101	0.92	2.00
East-5	West-5	1.36	9781.88
North-101	East-405	0.93	1.04
North-101	East-5	0.97	1.47
North-101	South-101	1.09	6.36
North-101	West-5	0.91	0.99
South-101	East-405	1.31	572.34
South-101	East-5	0.96	0.88
South-101	North-101	1.04	2.15
South-101	West-5	0.94	0.70
West-5	East-405	0.93	1.50
West-5	East-5	0.94	1.47
West-5	North-101	1.00	2.65
West-5	South-101	0.97	2.50

Figures

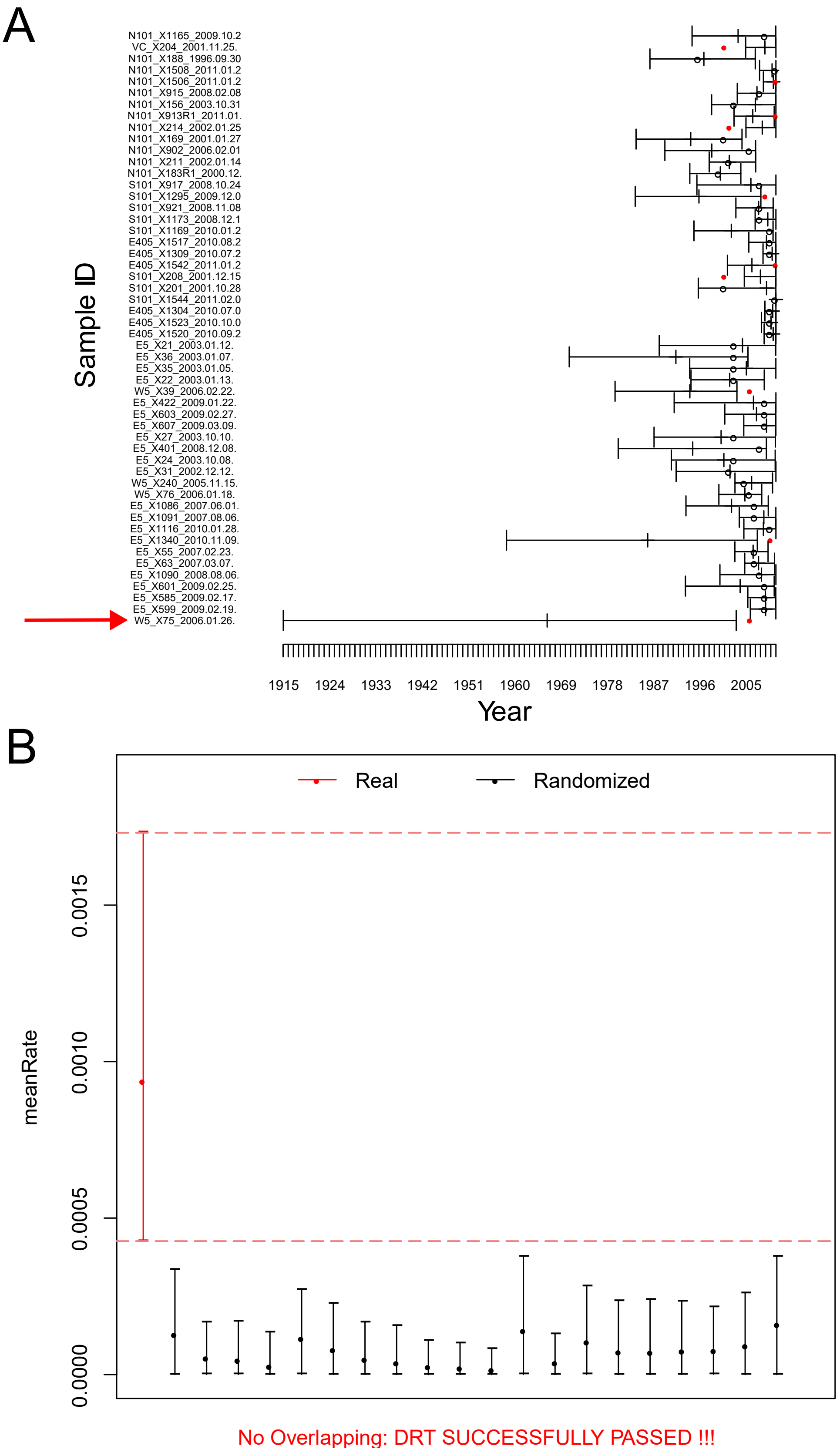

Figure S1. Test for temporal signal. A) Results from leave-one-out cross-validation identifying several samples where the sample date (red circle) occurs outside the 95% HPD

intervals for the estimated age of the sample, which suggests a given sequence may lead to biased temporal signal. One sequence, indicated by the red arrow, was subsequently removed from analysis. B) Results from the date randomization test performed on the meanRate BEAST parameter following removal of the indicated sequence. Non-overlap of 95% HPD intervals of the real dataset (red) with 20 datasets with randomized dates (black) indicates significant temporal signal.
